# Supplementary material for: Socioeconomic differences in participation and diagnostic yield within the Dutch national colorectal cancer screening programme with faecal immunochemical testing
Source: PLoS One. 2022 Feb 17;17(2):e0264067. doi: 10.1371/journal.pone.0264067 (PMC8853540; doi:10.1371/journal.pone.0264067)
Supplement: S1 Appendix — (PDF) [file pone.0264067.s001.pdf]

Appendix 1

Sensitivity analyses of all outcomes with deciles instead of deciles

Table 1: **Descriptive of the number, age and gender distribution of the invitees in each decile. Decile 1 least deprived, Decile 5 most deprived.**

			Gender			Age	
	**Number**	**%**	**Males**	**%**		**median**	
Decile 1	152290	8.2%	75653	49.7%		65.7	
Decile 2	181943	9.8%	90360	49.7%		65.7	
Decile 3	187725	10.1%	93555	49.8%		65.7	
Decile 4	193619	10.4%	96374	49.8%		65.9	
Decile 5	201600	10.8%	99731	49.5%		66.0	
Decile 6	202307	10.8%	100046	49.5%		65.9	
Decile 7	200609	10.8%	99064	49.4%		65.9	
Decile 8	188055	10.1%	92277	49.1%		66.7	
Decile 9	189994	10.2%	91373	48.1%		66.8	
Decile 10	167918	9.0%	80849	48.1%		66.8	
**Total**	1866060	100.0%	919282	49.3%	$p<0.001$	65.9	$p<0.001$

Table 2: **The participation to FIT, positivity rate and colonoscopy uptake after a positive FIT in each decile, with the univariate and multivariate odds ratio (OR) and 95% CI.**

Decile	N	Attendance to FIT	OR (univariate)	OR (multi-variate)*	95% CI	
Decile 1	110528	72.6%	1	1		p<0.0001
Decile 2	136330	74.9%	1.13	1.13	1.11 - 1.15	
Decile 3	141021	75.1%	1.14	1.14	1.13 - 1.16	
Decile 4	145506	75.2%	1.14	1.15	1.13 - 1.17	
Decile 5	151628	75.2%	1.15	1.15	1.14 - 1.17	
Decile 6	151505	74.9%	1.13	1.13	1.12 - 1.15	
Decile 7	147844	73.7%	1.06	1.07	1.05 - 1.08	
Decile 8	135796	72.2%	0.98	0.99	0.98 - 1.01	
Decile 9	131945	69.4%	0.86	0.87	0.86 - 0.88	
Decile 10	108000	64.3%	0.68	0.69	0.68 - 0.70	
	**N**	**Positivity rate**	**OR (univariate)**	**OR (multi-variate)***	**95% CI**	
Decile 1	6398	5.8%	1	1		p<0.0001
Decile 2	8068	5.9%	1.02	1.02	0.99 - 1.06	
Decile 3	8674	6.1%	1.07	1.06	1.03 - 1.10	
Decile 4	9052	6.2%	1.08	1.07	1.04 - 1.11	
Decile 5	9559	6.3%	1.10	1.09	1.05 - 1.12	
Decile 6	9676	6.4%	1.11	1.10	1.07 - 1.14	
Decile 7	9654	6.5%	1.14	1.13	1.09 - 1.16	
Decile 8	9383	6.9%	1.21	1.19	1.15 - 1.23	
Decile 9	9173	6.9%	1.22	1.20	1.16 - 1.24	
Decile 10	7972	7.3%	1.30	1.28	1.24 - 1.33	
	**N**	**Attendance to diagnostic colonoscopy**	**OR (univariate)**	**OR (multi-variate)***	**95% CI**	
Decile 1	5155	80.6%	1	1		p<0.0001
Decile 2	6613	82.0%	1.10	1.10	1.01 - 1.19	
Decile 3	7172	82.7%	1.15	1.15	1.06 - 1.25	
Decile 4	7440	82.2%	1.11	1.12	1.03 - 1.21	
Decile 5	7862	82.2%	1.12	1.12	1.04 - 1.22	
Decile 6	7870	81.3%	1.05	1.06	0.98 - 1.15	
Decile 7	7830	81.1%	1.04	1.04	0.96 - 1.13	
Decile 8	7404	78.9%	0.90	0.91	0.84 - 0.99	
Decile 9	7068	77.1%	0.81	0.82	0.76 - 0.89	
Decile 10	5924	74.3%	0.70	0.71	0.65 - 0.77	

* The multivariate OR is corrected for age and gender.

Table 3: **The positive predictive value (PPV) of FIT for advanced neoplasia (AN) and colorectal cancer (CRC) in each SES decile, with the univariate and multivariate odds ratio (OR) and 95% CI.**

	N	PPV AN*	OR (univariate)	OR (multi-variate)**	95% CI	
Decile 1	2863	55.5%	1	1		p<0.001
Decile 2	3826	57.9%	1.10	1.10	1.02 - 1.18	
Decile 3	4125	57.5%	1.08	1.08	1.00 - 1.16	
Decile 4	4263	57.3%	1.07	1.07	1.00 - 1.15	
Decile 5	4613	58.7%	1.14	1.13	1.06 - 1.22	
Decile 6	4578	58.2%	1.11	1.11	1.03 - 1.19	
Decile 7	4608	58.9%	1.14	1.15	1.07 - 1.23	
Decile 8	4264	57.6%	1.09	1.09	1.01 - 1.17	
Decile 9	4024	56.9%	1.06	1.06	0.99 - 1.14	
Decile 10	3271	55.2%	0.99	1.00	0.92 - 1.07	
	**N**	**PPV CRC***	**OR (univariate)**	**OR (multi-variate)****	**95% CI**	
Decile 1	481	9.3%	1	1		p=0.04
Decile 2	622	9.4%	1.01	1.01	0.89 - 1.14	
Decile 3	673	9.4%	1.01	1.00	0.89 - 1.14	
Decile 4	703	9.4%	1.01	1.01	0.89 - 1.14	
Decile 5	759	9.7%	1.04	1.03	0.91 - 1.16	
Decile 6	757	9.6%	1.03	1.03	0.91 - 1.16	
Decile 7	683	8.7%	0.93	0.92	0.82 - 1.04	
Decile 8	618	8.3%	0.88	0.87	0.77 - 0.99	
Decile 9	655	9.3%	0.99	0.98	0.86 - 1.11	
Decile 10	510	8.6%	0.92	0.90	0.79 - 1.03	

*An advanced adenoma was defined as any adenoma with histology showing ≥25% villous component or high-grade dysplasia or with size ≥10 mm. The PPV was calculated as the number of persons with an advanced adenoma or with a CRC (together called advanced neoplasia (AN) divided by the number of persons who underwent a colonoscopy after a positive FIT.

**The multivariate OR is corrected for age and gender.

Table 4: **The detection rate (DR) per 100 participants uncorrected and corrected for colonoscopy uptake and the yield per 100 invitees of advanced neoplasia (AN) and colorectal cancer (CRC) for each decile, with the univariate and multivariate odds ratio (OR) and 95% CI.**

		DR PER PARTICIPANT						YIELD PER INVITEE				
	N	DR AN uncorrected*	DR AN corrected**	OR (univariate)	OR (multi-variate)***	95% CI		yield AN	OR (univariate)	OR (multi-variate)***	95% CI	
Decile 1	2863	2.59%	3.21%	1	1		p<0.01	1.88%	1	1		p<0.0001
Decile 2	3826	2.81%	3.42%	1.09	1.07	1.02 - 1.11		2.10%	1.12	1.12	1.07 - 1.17	
Decile 3	4125	2.93%	3.53%	1.14	1.10	1.06 - 1.15		2.20%	1.17	1.17	1.11 - 1.22	
Decile 4	4263	2.93%	3.56%	1.14	1.11	1.07 - 1.16		2.20%	1.18	1.17	1.12 - 1.23	
Decile 5	4613	3.04%	3.70%	1.18	1.16	1.12 - 1.20		2.29%	1.22	1.21	1.16 - 1.27	
Decile 6	4578	3.02%	3.71%	1.18	1.16	1.12 - 1.20		2.26%	1.21	1.21	1.15 - 1.26	
Decile 7	4608	3.12%	3.84%	1.21	1.20	1.16 - 1.25		2.30%	1.23	1.22	1.16 - 1.28	
Decile 8	4264	3.14%	3.98%	1.22	1.25	1.21 - 1.29		2.27%	1.21	1.19	1.14 - 1.25	
Decile 9	4024	3.05%	3.96%	1.18	1.24	1.20 - 1.29		2.12%	1.12	1.11	1.06 - 1.17	
Decile 10	3271	3.03%	4.08%	1.16	1.28	1.24 - 1.33		1.95%	1.02	1.01	0.96 - 1.06	
	N	DR CRC*	DR CRC**	OR (univariate)	OR (multi-variate)***	95% CI		yield CRC	OR (univariate)	OR (multi-variate)***	95% CI	
Decile 1	481	0.44%	0.54%	1	1		p<0.01	0.32%	1	1		p<0.001
Decile 2	622	0.46%	0.56%	1.05	1.03	0.95 - 1.11		0.34%	1.08	1.08	0.96 - 1.22	
Decile 3	673	0.48%	0.58%	1.10	1.07	0.99 - 1.15		0.36%	1.14	1.13	1.01 - 1.27	
Decile 4	703	0.48%	0.59%	1.11	1.09	1.01 - 1.16		0.36%	1.15	1.14	1.01 - 1.28	
Decile 5	759	0.50%	0.61%	1.15	1.13	1.05 - 1.20		0.38%	1.19	1.18	1.05 - 1.32	
Decile 6	757	0.50%	0.62%	1.15	1.14	1.07 - 1.21		0.37%	1.19	1.17	1.05 - 1.31	
Decile 7	683	0.46%	0.57%	1.06	1.05	0.98 - 1.13		0.34%	1.08	1.06	0.95 - 1.20	
Decile 8	618	0.46%	0.58%	1.05	1.07	0.99 - 1.15		0.33%	1.04	1.02	0.91 - 1.15	
Decile 9	655	0.50%	0.65%	1.14	1.19	1.12 - 1.27		0.34%	1.09	1.07	0.95 - 1.20	
Decile 10	510	0.47%	0.64%	1.09	1.18	1.10 - 1.26		0.30%	0.96	0.94	0.83 - 1.06	

*An advanced adenoma was defined as any adenoma with histology showing ≥25% villous component or high-grade dysplasia or with size ≥10 mm. The detection rate was defined as the number of persons with advanced adenomas or with CRC (together called advanced neoplasia (AN)) detected during colonoscopy divided by the number of screened persons with an assessable stool sample.

**The detection rate was corrected for the differences in colonoscopy uptake compared to decile 1.

***The multivariate OR is corrected for age and gender and in the analysis per participant we corrected the DR for non-compliance to colonoscopy using poststratification (assuming full compliance).
