# Supplementary material for: Socioeconomic differences in participation and diagnostic yield within the Dutch national colorectal cancer screening programme with faecal immunochemical testing
Source: PLoS One. 2022 Feb 17;17(2):e0264067. doi: 10.1371/journal.pone.0264067 (PMC8853540; doi:10.1371/journal.pone.0264067)
Supplement: Attachment — Submitted filename: Response to reviewers comments_20220111.docx [file pone.0264067.s002.docx]

1. We note that the grant information you provided in the ‘Funding Information’ and ‘Financial Disclosure’ sections do not match.

We cross-checked the provided information. They naming did actually match, however, the inconsistency was in the language; the Dutch and the English name of the exact same institution.

When you resubmit, please ensure that you provide the correct grant numbers for the awards you received for your study in the ‘Funding Information’ section.

Please include your amended statements within your cover letter; we will change the online submission form on your behalf.

2. We note that you have included the phrase “data not shown” in your manuscript. Unfortunately, this does not meet our data sharing requirements. PLOS does not permit references to inaccessible data. We require that authors provide all relevant data within the paper, Supporting Information files, or in an acceptable, public repository. Please add a citation to support this phrase or upload the data that corresponds with these findings to a stable repository (such as Figshare or Dryad) and provide and URLs, DOIs, or accession numbers that may be used to access these data. Or, if the data are not a core part of the research being presented in your study, we ask that you remove the phrase that refers to these data.

Indeed, one sentence in the discussion relied on data not shown. Our feeling is that adding the data itself is not in proportion to what the sentence adds to the paper. Therefore, the following sentence can be omitted from the paper:

"However, we did not find a difference in ORs for colonoscopy uptake if we corrected for the individuals that were excluded for colonoscopy at intake (data not shown)."

3. Your ethics statement should only appear in the Methods section of your manuscript. If your ethics statement is written in any section besides the Methods, please move it to the Methods section and delete it from any other section. Please ensure that your ethics statement is included in your manuscript, as the ethics statement entered into the online submission form will not be published alongside your manuscript.

We moved the ethics statement to the Methods.

4. Please include your tables as part of your main manuscript and remove the individual files. Please note that supplementary tables (should remain/ be uploaded) as separate "supporting information" files.

Tables were added to the Main Manuscript.

5. In the ethics statement in the manuscript methods and the online submission form, please state whether the authors were involved in data collection, or had access to any identifying patient information at any time. Please also provide additional details about what information patients were provided about the use of their data for scientific research, and how they were able to opt out.

We added the relevant additional information.

*“The authors note that the sharing of the speudonymised dataset is not allowed. Researchers had access to the data for the purpose of evaluation of the screening programme. The data is owned by the BVO-NL. All data of the national CRC screening programme is stored in the national database ScreenIT. Data of this study as well as other data of the national CRC screening programme are only available with the permission of BV_NL. Researchers interested in accessing and analysing data of the national CRC screening database ScreenIT may contact the data officer of BVO-NL (wetenschappelijkonderzoek@fsb-ssc.nl).*

*Data is obtained through the population based national screening programme..\ Permit for carrying out the national screening programme is laid down in the Population Screening Act. The researcher were not involved in data collection and had only access to pseudonymised individual level data By participating individuals gave their implied informed consent. All individuals could object to use their data for scientific research.”*

6. Please upload a Response to Reviewers letter which should include a point by point response to each of the points made by the Editor and / or Reviewers. (This should be uploaded as a 'Response to Reviewers' file type.) Please follow this link for more information: [https://eur01.safelinks.protection.outlook.com/?url=http%3A%2F%2Fblogs.plos.org%2Feveryone%2F2011%2F05%2F10%2Fhow-to-submit-your-revised-manuscript%2F&amp;data=04%7C01%7Ce.toes-zoutendijk%40erasmusmc.nl%7C371ecd3ba3d14a5b268608d9d17d1eb2%7C526638ba6af34b0fa532a1a511f4ac80%7C0%7C0%7C637771157606594658%7CUnknown%7CTWFpbGZsb3d8eyJWIjoiMC4wLjAwMDAiLCJQIjoiV2luMzIiLCJBTiI6Ik1haWwiLCJXVCI6Mn0%3D%7C3000&amp;sdata=LLvlZXAXUse6UZa4%2Bzh%2FaIC4Iu5IW5buXUrSu5p5wDo%3D&amp;reserved=0](https://eur01.safelinks.protection.outlook.com/?url=http%3A%2F%2Fblogs.plos.org%2Feveryone%2F2011%2F05%2F10%2Fhow-to-submit-your-revised-manuscript%2F&data=04%7C01%7Ce.toes-zoutendijk%40erasmusmc.nl%7C614cac70422d4eaed44d08d9d1db93b7%7C526638ba6af34b0fa532a1a511f4ac80%7C0%7C0%7C637771563864649785%7CUnknown%7CTWFpbGZsb3d8eyJWIjoiMC4wLjAwMDAiLCJQIjoiV2luMzIiLCJBTiI6Ik1haWwiLCJXVCI6Mn0%3D%7C3000&sdata=%2Bw2Rg23QJtj3HRKcSe%2B%2Fk5uQWqr8kLMhO5HUZDmUgf8%3D&reserved=0)


7. Please amend your list of authors on the manuscript to ensure that each author is linked to an affiliation.
We note that you have included affiliation numbers 1,2,3,4,5,6,7 and 8however only affiliations 1,2,3,4,5,6,7 and 9 have authors linked to them. Please amend affiliation 8 to link an author to it or remove if added in error.

Apologizies fort his error, we changed it.

8. We note your current Data Availability Statement: “Data cannot be shared publicly because of third party data. Data underlying the results presented in the study are stored in the national screening database ScreenIT, owned bij FSB."

For studies involving third-party data, we encourage authors to share any data specific to their analyses that they can legally distribute. PLOS recognizes, however, that authors may be using third-party data they do not have the rights to share. When third-party data cannot be publicly shared, authors must provide all information necessary for interested researchers to apply to gain access to the data. ([https://eur01.safelinks.protection.outlook.com/?url=https%3A%2F%2Fjournals.plos.org%2Fplosone%2Fs%2Fdata-availability%23loc-acceptable-data-access-restrictions&amp;data=04%7C01%7Ce.toes-zoutendijk%40erasmusmc.nl%7C371ecd3ba3d14a5b268608d9d17d1eb2%7C526638ba6af34b0fa532a1a511f4ac80%7C0%7C0%7C637771157606594658%7CUnknown%7CTWFpbGZsb3d8eyJWIjoiMC4wLjAwMDAiLCJQIjoiV2luMzIiLCJBTiI6Ik1haWwiLCJXVCI6Mn0%3D%7C3000&amp;sdata=C07RWjs4IEkje38QRbO1C1Yr4jr78%2F1lcvMcXOmLe34%3D&amp;reserved=0](https://eur01.safelinks.protection.outlook.com/?url=https%3A%2F%2Fjournals.plos.org%2Fplosone%2Fs%2Fdata-availability%23loc-acceptable-data-access-restrictions&data=04%7C01%7Ce.toes-zoutendijk%40erasmusmc.nl%7C614cac70422d4eaed44d08d9d1db93b7%7C526638ba6af34b0fa532a1a511f4ac80%7C0%7C0%7C637771563864649785%7CUnknown%7CTWFpbGZsb3d8eyJWIjoiMC4wLjAwMDAiLCJQIjoiV2luMzIiLCJBTiI6Ik1haWwiLCJXVCI6Mn0%3D%7C3000&sdata=CT%2FkDpUrRX5MvQma8vaLUshB37k7zWwMjnt%2BI7qEM%2BM%3D&reserved=0))

For any third-party data that the authors cannot legally distribute, they should include the following information in their Data Availability Statement upon submission:
1. A description of the data set and the third-party source
2. If applicable, verification of permission to use the data set
3. Confirmation of whether the authors received any special privileges in accessing the data that other researchers would not have
4. All necessary contact information others would need to apply to gain access to the data

The researchers had access to the data as part of their role as the party responsible for the national evaluation of the Dutch colorectal cancer programme. Data is owned by the FSB and it not available to all researchers. Researchers interested in using data from the national colorectal cancer screening database may request access FSB. We updated the statement in the manuscript.

*“The authors note that the sharing of the speudonymised dataset is not allowed. Researchers had access to the data for the purpose of evaluation of the screening programme. The data is owned by the samenwerking bevolkingsonderzoeken (FSB). All data of the national CRC screening programme is stored in the national database ScreenIT. Data of this study as well as other data of the national CRC screening programme are only available with the permission of FSB. Researchers interested in accessing and analysing data of the national CRC screening database ScreenIT may contact the corresponding author.*

*Data is obtained through the population based national screening programme.. Permit for carrying out the national screening programme is laid down in the Population Screening Act. The researcher were not involved in data collection and had only access to pseudonymised individual level data By participating individuals gave their implied informed consent. All individuals could object to use their data for scientific research.”*
